# Orthostatic hypotension is associated with higher levels of circulating endostatin

**DOI:** 10.1093/ehjopen/oeae030

**Published:** 2024-04-10

**Authors:** Fabrizio Ricci, Anders Larsson, Toralph Ruge, Kristian Galanti, Viktor Hamrefors, Richard Sutton, Brian Olshansky, Artur Fedorowski, Madeleine Johansson

**Affiliations:** Department of Clinical Sciences, Lund University, Malmö, Sweden, Jan Waldenströms gata 35, 214 28 Malmö, Sweden; Department of Neuroscience, Imaging and Clinical Sciences, ‘G.d'Annunzio’ University of Chieti-Pescara, Chieti, Italy; Heart Department, ‘SS Annunziata’ Polyclinic University Hospital, Chieti, Italy; Section of Clinical Chemistry, Department of Medical Sciences, Uppsala University, Uppsala, Sweden; Department of Clinical Sciences, Lund University, Malmö, Sweden, Jan Waldenströms gata 35, 214 28 Malmö, Sweden; Department of Emergency and Internal Medicine, Skåne University Hospital, Malmö, Sweden; Department of Neuroscience, Imaging and Clinical Sciences, ‘G.d'Annunzio’ University of Chieti-Pescara, Chieti, Italy; Department of Clinical Sciences, Lund University, Malmö, Sweden, Jan Waldenströms gata 35, 214 28 Malmö, Sweden; Department of Cardiology, Skåne University Hospital, Malmö, Sweden, Jan Waldenströms gata 15, 214 28 Malmö, Sweden; Department of Cardiology, Hammersmith Hospital, National Heart and Lung Institute, Imperial College, London, UK; Division of Cardiology, Department of Internal Medicine, University of Iowa Hospitals and Clinics, Iowa City, USA; Department of Clinical Sciences, Lund University, Malmö, Sweden, Jan Waldenströms gata 35, 214 28 Malmö, Sweden; Department of Cardiology, Karolinska University Hospital, Stockholm, Sweden; Department of Medicine, Karolinska Institute, Stockholm, Sweden; Department of Clinical Sciences, Lund University, Malmö, Sweden, Jan Waldenströms gata 35, 214 28 Malmö, Sweden; Department of Cardiology, Skåne University Hospital, Malmö, Sweden, Jan Waldenströms gata 15, 214 28 Malmö, Sweden

**Keywords:** Orthostatic hypotension, Endostatin, Blood pressure

## Abstract

**Aims:**

The pathophysiology of orthostatic hypotension (OH), a common clinical condition, associated with adverse outcomes, is incompletely understood. We examined the relationship between OH and circulating endostatin, an endogenous angiogenesis inhibitor with antitumour effects proposed to be involved in blood pressure (BP) regulation.

**Methods and results:**

We compared endostatin levels in 146 patients with OH and 150 controls. A commercial chemiluminescence sandwich immunoassay was used to measure circulating levels of endostatin. Linear and multivariate logistic regressions were conducted to test the association between endostatin and OH. Endostatin levels were significantly higher in OH patients (59 024 ± 2513 pg/mL) vs. controls (44 090 ± 1978pg/mL, *P* < 0.001). A positive linear correlation existed between endostatin and the magnitude of systolic BP decline upon standing (*P* < 0.001). Using multivariate analysis, endostatin was associated with OH (adjusted odds ratio per 10% increase of endostatin in the whole study population = 1.264, 95% confidence interval 1.141–1.402), regardless of age, sex, prevalent cancer, and cardiovascular disease, as well as traditional cardiovascular risk factors.

**Conclusion:**

Circulating endostatin is elevated in patients with OH and may serve as a potential clinical marker of increased cardiovascular risk in patients with OH. Our findings call for external validation. Further research is warranted to clarify the underlying pathophysiological mechanisms.

## Graphical Abstract

**Graphical Abstract:**
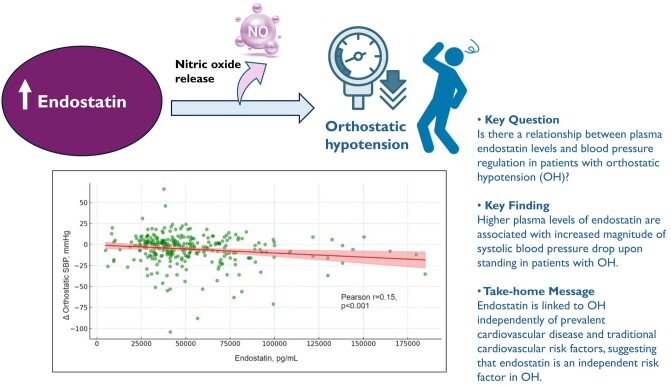

## Introduction

Idiopathic orthostatic hypotension (OH), defined as a systolic blood pressure (SBP) drop of ≥20 mmHg and/or diastolic BP drop of ≥10 mmHg within 3 min of standing,^1^ can occur when BP regulation fails.

We have previously shown that OH is linked to a range of dysregulated molecular events, such as higher plasma concentration of various cardiovascular and inflammatory biomarkers, in particular matrix metalloproteinase (MMP)-7, thrombomodulin, T cell immunoglobulin, immunoglobulin-like transcript 3, midkine, and regenerating islet-derived protein 4, independently of age, sex, prevalent cardiovascular disease, and risk factors.^2,3^ Yet, the underlying molecular mechanism of OH remains largely unexplored.

Endostatin is a biologically active peptide cleaved by MMPs, elastases, and cathepsins from collagen XVIII in the extracellular matrix.^4,5^ It suppresses tumour formation through the inhibition of blood vessel growth (angiogenesis) and has emerged as a potential anticancer therapeutic agent.^6,7^ Previous studies have shown that elevated circulating endostatin is linked to different manifestations of cardiovascular diseases, such as heart failure, coronary artery disease, myocardial infarction, and ischaemic stroke,^8–13^ as well as hypertensive target organ damage^14^ and cardiovascular mortality.^15^ Data suggest that higher circulating levels of endostatin reduce BP by inducing nitric oxide (NO) release,^16,17^ resulting in vasodilation, suggesting that endostatin may be an important regulator of vascular tone.

Considering that OH is associated with incident cardiovascular disease (CVD),^18^ and circulating endostatin is linked to increased risk of CVD, we tested the hypothesis whether a pathophysiological connection between OH and endostatin exists. We compared circulating levels of endostatin in patients who were diagnosed with OH using head-up tilt (HUT) test with controls.

## Methods

### Study population and design

In the present study, a prospective case–control design was utilized to explore the relationship between circulating endostatin levels and the presence of OH. The Syncope Study of Unselected Population in Malmö (SYSTEMA) cohort was initiated in 2008.^19^ Patients with unexplained syncope and/or symptoms of orthostatic intolerance were referred to the tertiary syncope unit at Skåne University Hospital in Malmö from outpatient care and hospitals in Sweden. Additional tests were performed whenever indicated to exclude any cardiac and neurological causes of symptoms, e.g. exercise test, ambulatory prolonged electrocardiogram (Holter ECG), 2D transthoracic echocardiography, coronary and pulmonary angiography, brain imaging, and encephalography. During the study period, over 3000 patients were examined by HUT according to current European syncope guidelines.^1^

In this case–control study, we included 146 patients, randomly selected, with OH verified by a positive HUT test, and defined as a SBP drop of ≥20 mmHg and/or diastolic BP drop of ≥10 mmHg within 3 min of standing,^1^ and compared them with 150 controls from SYSTEMA with a negative HUT test. Data on baseline characteristics, such as diabetes, cancer, cardiovascular disease, and stroke, were self-reported.

### Ethical approval

All study participants gave written informed consent. The study protocol conformed to the ethical guidelines of the 1975 Declaration of Helsinki and its later amendments and was approved by The Regional Ethical Review Board of Lund University (No. 82/2008).

### Examination protocol

Patients were taking their regular medications and fasted for 2 h before examination but were allowed to drink water at will. They were asked to fill out a questionnaire about past medical history. The patients were placed on a tilt table and rested for at least 10 min before blood samples were collected through a venous cannula inserted in the forearm. Subsequently, patients rested for another 10 min to obtain haemodynamically stable parameters; thereafter, the standardized 70° HUT was carried out for 20 min followed by nitroglycerine provocation according to the Italian protocol if passive HUT was negative or until syncope/pre-syncope or pronounced symptoms of orthostatic intolerance occurred.^20^ Continuous beat-to-beat BP and ECG monitoring was performed by a validated non-invasive photoplethysmographic method (Nexfin monitor, BMEYE, Amsterdam, The Netherlands, or Finapres Nova, Finapres Medical Systems, Enschede, The Netherlands) utilizing a wrist unit and finger cuff of appropriate size. Classical OH was defined as a sustained BP fall within 3 min of HUT, according to the international consensus diagnostic criteria for OH, i.e. SBP drop ≥20 mmHg or DBP drop ≥10 mmHg; SBP drop ≥30 mmHg if supine SBP ≥160 mmHg; or sustained SBP ≤90 mmHg during HUT.^21^

### Measurement of endostatin

Plasma endostatin was measured from supine blood samples (total volume: 30 mL) that had been first centrifuged, then stored as 16 × 250 μL aliquots of EDTA plasma in plastic thermotubes, and frozen at −80°C. For endostatin analysis, the samples were thawed and examined by sandwich immunoassay using a commercially available ELISA kit for endostatin (DY1098, R&D Systems, Minneapolis, MN) according to the manufacturer’s instructions. The assays had a total coefficient of variation of ≈7%.^14^ The endostatin assay has been highly validated for accurate quantitation and long-term reproducibility and has been previously used in other patient populations.^11,14,22,23^ R&D System has documented the specificity and cross-reactivity of the antibodies against a broad range of cytokines in their Quantikine version of the assay. No significant cross-reactivity or interference has been observed.

### Power calculation

To determine the appropriate sample size, a power analysis was performed prior to data collection. We anticipated a medium effect size (Cohen’s *d* = 0.5) for the difference in endostatin levels between OH patients and controls, based on clinical relevance and previously observed effect sizes in related cardiovascular biomarker studies. This effect size reflects a balance between detectability and clinical significance, where a medium effect size is substantial enough to be of interest for potential future clinical applications and interventions.

For our primary analysis, an independent samples *t*-test was chosen to compare mean plasma endostatin levels between the groups. We set a significance level (alpha) at 0.05 and aimed for a study power of 80% (1—beta), to ensure a high probability of detecting a true effect, if present. According to the power analysis using the G*Power software, the required sample size to detect a medium effect size at the given power and significance level was calculated to be ∼128 participants (64 in each group). However, to account for possible attrition and non-normality of distribution, we increased our sample size by 20%, resulting in a total sample size of 154 participants. The actual sample size in the study was 296 participants, which exceeds the calculated requirement. This larger sample size increases the study’s ability to detect even smaller effect sizes, thereby enhancing the robustness of the findings. Given the final sample size of 296 participants, the study was ultimately well powered for detecting clinically meaningful differences in circulating endostatin levels between groups, also providing a stronger basis for the multivariate logistic regression analysis, which adjusted for potential confounders, further confirming the association of endostatin levels with OH.

### Statistical analysis

Baseline characteristics of the study participants were summarized and compared by OH status (OH+ vs. OH−). Categorical variables were expressed as frequencies and percentages and compared using the *χ*^2^ test or Fisher’s exact test, as appropriate. Continuous variables were described using mean ± standard deviation (SD) or median with interquartile range (IQR), depending on their distribution, and compared using Student’s *t*-test or the Mann–Whitney *U* test. Endostatin levels, measured in picograms per millilitre, were compared between OH+ and OH− groups using an independent samples *t*-test or Mann–Whitney *U* test, depending on the distribution of the data. The normality of data was assessed using the Shapiro–Wilk test. To test the degree of linear relationship between circulating endostatin levels and the magnitude of the orthostatic change in SBP adjusted for baseline supine SBP, data were visually inspected using a scatter plot; the Pearson correlation coefficient was computed. We also assessed the correlation between plasma levels of endostatin stratified according to tertiles of orthostatic SBP drop during HUT using the Kruskal–Wallis test.

To further assess the individual effect of orthostatic SBP drop on circulating endostatin levels adjusted for potential confounders, such as baseline supine SBP, age, and antihypertensive treatment, we performed a multiple linear regression model adjusted for the abovementioned confounders. To assess how the relationship between endostatin and orthostatic SBP drop might vary with changes in baseline supine SBP, age, and antihypertensive treatment, non-linear relationships and interaction effects were assessed by a polynomial transformation applied to endostatin levels and interaction terms between endostatin and predictors. Model fit was assessed using the coefficient of determination (*R*^2^), providing a measure of the variance in orthostatic SBP drop explained by the models. These metrics facilitated comparison between the linear and non-linear models, aiding in the identification of the most appropriate model for capturing the relationships within the data.

A multivariate logistic regression model was employed to identify factors independently associated with the presence of OH. The model was adjusted for potential confounders identified based on their clinical relevance and statistical significance in univariate analyses. The results were reported as odds ratios (ORs) with 95% confidence intervals (CIs). The goodness of fit for the logistic regression model was evaluated using the Hosmer–Lemeshow test. All covariates were incorporated into the same logistic regression model. This comprehensive model was constructed to adjust for potential confounders to accurately determine the independent association between endostatin levels and OH.

Receiver operating characteristic (ROC) curve analysis was performed to evaluate the diagnostic performance of endostatin levels in predicting OH. The area under the ROC curve (AUC) was calculated, and the optimal cut-off value for endostatin was determined based on the Youden index. Sensitivity and specificity were reported for the best cut-off obtained. All tests were two tailed. A *P* < 0.05 was considered statistically significant. The statistical analysis was conducted using Wizard 2 (version 2.0.16), Python, leveraging libraries such as Pandas for data manipulation, Statsmodels for regression analyses, and Matplotlib for data visualization.

## Results

Baseline characteristics are reported in *Table 1*. The mean age differed significantly between patients with OH (59.8 ± 16.7 years) and the control group (52.3 ± 17.2 years, *P* < 0.001). Out of all OH patients, 91% were symptomatic and had a history of syncope. Sex distribution was comparable (OH: 47% women vs. controls: 49% women, *P* = 0.721). Increased prevalence of cancer (*P* < 0.001), stroke (*P* = 0.013), and antihypertensive medications (*P* = 0.003) were also found in OH. Notably lower minimum orthostatic systolic (95 ± 20 vs. 125 ± 18 mmHg, *P* < 0.001) and diastolic BP (61 ± 13 vs. 77 ± 10 mmHg, *P* < 0.001) were observed in OH compared with controls, as expected.

**Table 1:** Baseline characteristics of the study population (*n* = 296)

	OH, *n* = 146	Controls, *n* = 150	*P*-value
Age, years (age range)	59.8 ± 16.7	52.3 ± 17.2	**<0**.**001**
Sex, women, *n* (%)	69 (47)	74 (49)	0.721
BMI, kg/m^2^	25.9	25.6	0.269
eGFR, mL/min/1.73 m^2^	83 ± 23	78 ± 16	**0**.**029**
Current smoking, *n* (%)	19 (14)	18 (13)	0.786
Blood pressure parameters			
Supine HR, b.p.m.	68 ± 11	69 ± 12	0.864
Supine SBP, mmHg	138 ± 22	134 ± 19	0.050
Supine DBP, mmHg	75 ± 12.0	75 ± 10	0.470
Max orthostatic HR, b.p.m.	84 ± 15	82 ± 14	0.298
Min orthostatic SBP, mmHg	95 ± 20	125 ± 18	**<0**.**001**
Min orthostatic DBP, mmHg	61 ± 13	77 ± 10	**<0**.**001**
Past medical history and treatment			
Antihypertensive treatment, *n* (%)	56 (38.0)	34 (23.0)	**0**.**003**
Diabetes, *n* (%)	9 (6.2)	10 (6.7)	0.860
Stroke, *n* (%)	17 (11.7)	6 (4.0)	**0**.**013**
Ischaemic heart disease, *n* (%)	14 (9.7)	10 (6.7)	0.348
Heart failure, *n* (%)	6 (4.0)	2 (1.0)	0.138
Cancer, *n* (%)	29 (20.0)	9 (6.0)	**<0**.**001**

Continuous variables were reported as mean ± SD and *n* (%) for categorical variables. The bold values indicate a statistically significant *P*-value <0.05.

b.p.m., beats per minute; BMI, body mass index; DBP, diastolic blood pressure; eGFR, estimated glomerular filtration rate; HR, heart rate; min, minimum; max, maximum; OH, orthostatic hypotension; SBP, systolic blood pressure.

**Figure 1:**
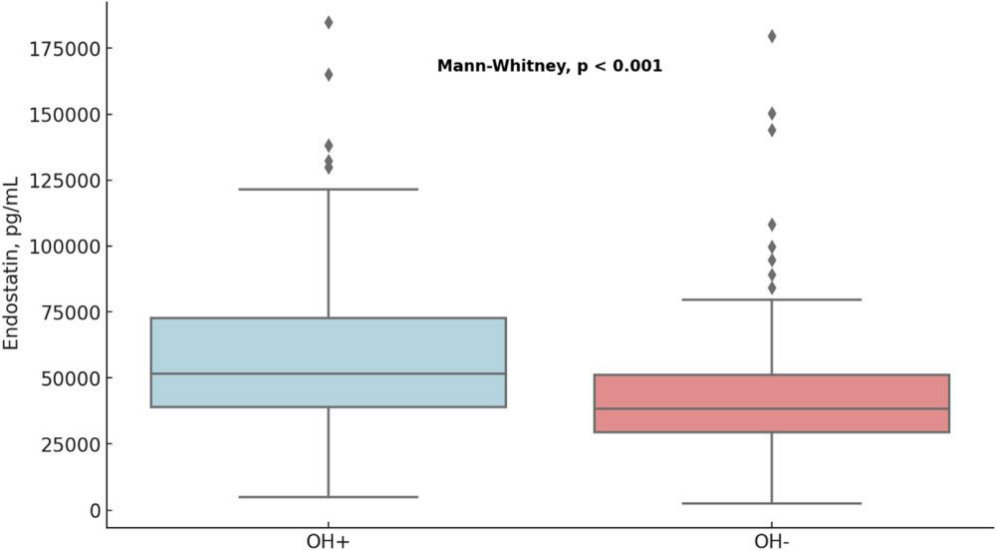
Comparison of the distribution of plasma endostatin levels in patients with orthostatic hypotension vs. controls (*n* = 296). The box and whisker plot illustrates the distribution of endostatin levels across orthostatic hypotension-positive (left) and orthostatic hypotension-negative (right) groups. The plot reveals median, quartiles, and outliers within each group. Our findings indicate that endostatin levels are significantly higher in patients with orthostatic hypotension than in the control group (*P* < 0.001).

**Figure 2:**
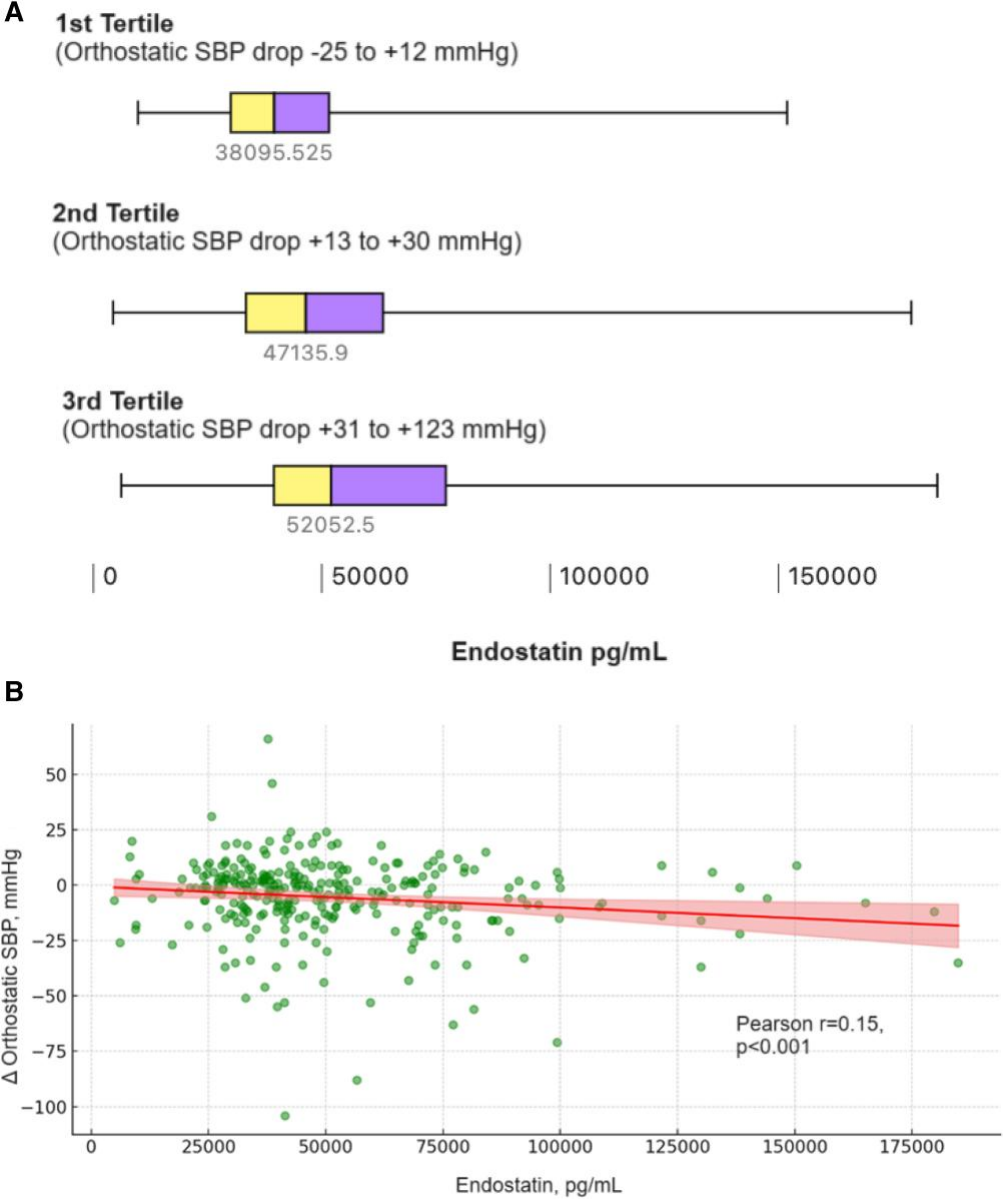
(*A*) Correlation between plasma endostatin levels and tertiles of orthostatic systolic blood pressure drop. The correlation between plasma endostatin levels stratified according to tertiles of orthostatic systolic blood pressure drop during head-up tilt. Boxplots represent pooled population, i.e. both orthostatic hypotension and controls. A negative systolic blood pressure value indicates an increase in systolic blood pressure upon standing, and a positive value systolic blood pressure reduction. Some subjects in the first tertile demonstrated increased systolic blood pressure upon standing, i.e. orthostatic hypertension, as such their orthostatic systolic blood pressure increased up to 25 mmHg, while in others it dropped by 12 mmHg. As demonstrated, levels of circulating endostatin were significantly higher across increasing tertiles of orthostatic systolic blood pressure drop, i.e. the higher the drop in systolic blood pressure, the higher the endostatin level (Kruskal–Wallis, *P* < 0.001). (*B*) Relationship between plasma endostatin levels and magnitude of orthostatic systolic blood pressure change. The scatter plot illustrates the relationship between plasma endostatin levels (measured in pg/mL) and the magnitude of orthostatic drop in systolic blood pressure (measured in mmHg), adjusted for baseline supine systolic blood pressure. Each dot represents individual patient data, indicating their endostatin level and the corresponding orthostatic systolic blood pressure drop. The regression line indicates a positive but weak correlation (Pearson’s *r* = 0.15, *P* < 0.001), suggesting that higher endostatin levels are associated with a greater drop in orthostatic systolic blood pressure.

Patients with OH exhibited significantly elevated levels of circulating endostatin compared with controls (59 024 ± 2513 vs. 44 090 ± 1978 pg/mL, *P* < 0.001; *Figure 1*). Plasma endostatin levels increased gradually across all tertiles of orthostatic SBP drop, i.e. the larger the drop in orthostatic SBP, the higher the endostatin level (Kruskal–Wallis, *P* < 0.001; *Figure 2A*). After adjusting for supine SBP, a significant yet modest positive linear correlation was observed between increasing levels of circulating endostatin and the magnitude of orthostatic SBP reduction upon standing (Pearson’s *r* = 0.15, *P* < 0.001; *Figure 2B*).

The extended linear and non-linear regression models indicated that baseline supine SBP, age, and antihypertensive treatment each were significantly inversely associated with orthostatic SBP drop and endostatin levels (see Supplementary material online, *Figures S1* and *S2*). The models explained 24 and 27% of the variance in orthostatic SBP drop, respectively (see Supplementary material online, *Table S1*). The interaction effect between baseline supine SBP and circulating endostatin levels on predicted orthostatic SBP drop was marginally significant (*P* for interaction 0.046; Supplementary material online, *Figure S3*), indicating that the effect of endostatin may vary depending on baseline supine SBP levels.

Using multivariate logistic regression analysis, after adjusting for clinically relevant confounders [including age, sex, body mass index (BMI), supine BP, antihypertensive medications, prevalent cardiovascular disease, cardiovascular risk factors, and cancer], endostatin remained independently associated with OH (adjusted OR per 10% increase of endostatin in the whole study population = 1.26, 95% CI 1.14–1.40, *P* < 0.001; *Table 2*). In a sensitivity analysis excluding cancer patients from the cohort, endostatin yielded an adjusted OR of 1.34 (95% CI 1.19–1.50, *P* < 0.001).

**Table 2:** Multivariate logistic regression analysis of factors associated with orthostatic hypotension

Covariate	Adjusted odds ratio	95% CI	*P*-value
Endostatin^a^	1.26	1.14–1.40	<0.001
Age	1.01	0.99–1.03	0.56
Sex, women	0.58	0.31–1.11	0.10
BMI	0.95	0.89–1.01	0.07
eGFR	1.00	0.98–1.02	0.90
Current smoking	1.09	0.67–1.78	0.72
Blood pressure parameters			
Supine HR	0.99	0.97–1.02	0.61
Supine SBP	1.00	0.98–1.02	0.98
Supine DBP	0.99	0.95–1.02	0.50
Past medical history and treatment			
Antihypertensive treatment	1.76	0.88–3.53	0.11
Diabetes	0.60	0.20–1.87	0.38
Stroke	2.24	0.74–6.75	0.15
Ischaemic heart disease	0.60	0.20–1.77	0.36
Cancer	3.60	1.50–8.65	0.004

b.p.m., beats per minute; BMI, body mass index; DBP, diastolic blood pressure; eGFR, estimated glomerular filtration rate; HR, heart rate; OH, orthostatic hypotension; SBP, systolic blood pressure.

^a^After removing cancer patients (*n* = 38), endostatin yielded an adjusted odds ratio of 1.34 (95% CI 1.193–1.504, *P* < 0.001) per 10% increase of endostatin in the whole study population.

**Figure 3:**
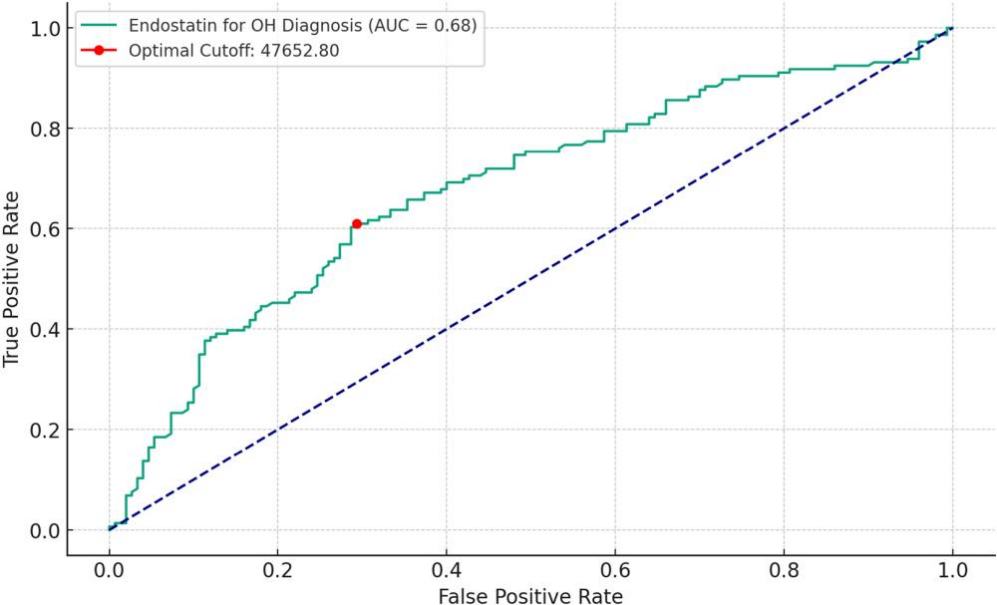
Receiver operating characteristic analysis of plasma endostatin levels for predicting orthostatic hypotension. The receiver operating characteristic curve demonstrates the sensitivity and specificity of endostatin levels at various thresholds. The optimal cut-off value for endostatin was 47 652.8 pg/mL (marked by the dot) yielding a sensitivity of 61% and specificity of 71%. The area under the curve was 0.68, indicating a good discriminative power of endostatin in predicting orthostatic hypotension.

Using ROC analysis, the optimal cut-off value of endostatin to predict OH was 47 652.8 pg/mL, yielding a discriminative power of AUC = 0.68, *P* < 0.001, with a sensitivity of 61% and a specificity of 71% (*Figure 3*).

## Discussion

In this study, we observed that patients with OH had significantly increased plasma levels of endostatin compared with controls. Notably, circulating endostatin levels were positively associated with OH independent of age, sex, and prevalent cardiovascular disease and cancer.

### Endostatin and cardiovascular risk in orthostatic hypotension

Endostatin is an endogenous protein that is cleaved from collagen XVIII by enzymes located in the extracellular matrix, including various MMPs^24,25^ that result in strong inhibition of angiogenesis and vascular endothelial growth factor (VEGF) expression (a key mediator of angiogenesis in cancer).^26^

Several studies have shown that MMPs may exacerbate arterial stiffness and endothelial dysfunction, resulting in increased CVD susceptibility.^27–29^ Arterial stiffness is independently associated with OH,^30^ and BP responses to orthostatic challenge, both BP reductions and BP increases, are independently and inversely associated with markers of aortic stiffness.^31^ Circulating levels of MMP-7 are independently associated with OH^2^ and involved in CVD promotion.^32,33^ Of note, our results showed that endostatin was linked to OH independently of prevalent cardiovascular disease and traditional cardiovascular risk factors, suggesting that endostatin is an independent risk factor in OH.

### Increased endostatin levels in orthostatic hypotension independently of prevalent cancer

Previous studies have shown that endostatin is a potential novel anticancer therapeutic agent that decreases tumour growth and prevents metastasis through inhibition of blood vessel formation, i.e. angiogenesis.^6,34^ Increased circulating endostatin is found in many human cancer forms,^35^ although the net effect on tumour angiogenesis seems to be determined by the balance between anti- and pro-angiogenic factors.^7^ Interestingly, despite increased prevalence of cancer and higher levels of circulating endostatin in patients with OH, endostatin was positively associated with OH independently of cancer, suggesting increased antiangiogenic activity in this patient group. A common side effect of treatment with inhibitors of angiogenesis is arterial hypertension,^36^ and Sunshine *et al.*^16^ proposed that co-administration of endostatin during antiangiogenesis therapy, e.g. VEGF inhibitors, may prevent the drug-induced hypertension.

### Endostatin and blood pressure regulation in orthostatic hypotension

Patients with OH have an impaired capacity to increase vascular resistance during standing, leading to increased pooling of venous blood in lower limbs.^37^ Nitric oxide is a potent vasodilator that plays a critical role in BP control.^38,39^ Previous studies have shown that higher circulating levels of endostatin reduce BP by inducing NO release,^16^ resulting in vasodilation. Endostatin causes a decrease in vascular tone through the endothelial NO synthase/soluble guanylyl cyclase (sGC) signalling pathway.^17^ Several studies support the role of NO/sGC in vasodilation and vascular dysfunction, which has enabled the development of potent and efficient modulators of sGC activity.^40^ We hypothesized that endostatin may play two regulatory roles in patients with OH. Firstly, endostatin may lower BP by NO release and vasodilation reducing the venous return. Secondly, endostatin may impair vascular tone. It has been shown that patients with autonomic failure have excess NO function that contributes to development of OH,^41^ although it remains unclear whether this results from increased NO production or sensitivity.

Interestingly, higher levels of circulating endostatin are associated with a disturbed circadian BP pattern in patients with type 2 diabetes, with significantly higher circulating endostatin levels in ‘non-dippers’ (i.e. patients with reduced decline in nocturnal BP) compared with ‘dippers’ (i.e. normal dipping pattern characterized by a >10% dip in the BP during the night).^42^ According to Wuopio *et al.*,^42^ these findings suggest that higher levels of circulating endostatin seen among ‘non-dippers’ may reflect extracellular remodelling due to damage in the kidneys, heart, and vascular tree. Disturbed circadian patterns of BP have also been reported in OH, with a higher prevalence of ‘non-dippers’ and ‘reverse dippers’ (i.e. patients with higher night-time BP compared with daytime BP).^43,44^ Further studies are needed to assess the relationship between endostatin and 24-h ambulatory BP monitoring in patients with OH.

The relatively weak correlation between orthostatic SBP change and endostatin when adjusted only for baseline supine SBP suggests that the linear model did not capture a large portion of the variance in SBP drop and that other factors may influence the SBP drop. As such, we extended our analyses, accounting for additional possible confounders, such as age and antihypertensive treatment, that resulted in stronger correlations. Furthermore, there was a marginally significant interaction effect between endostatin levels and baseline supine SBP on predicted orthostatic SBP drop, supporting the hypothesis that endostatin and baseline SBP might interact to influence SBP regulation. It also emphasizes the need for a multifactorial approach, accounting for both biomarkers such as endostatin and clinical measures such as baseline supine SBP, when studying determinants of orthostatic SBP drop in OH. External validation could provide deeper insights into these dynamics and their implications for understanding and treating OH.

### Clinical relevance

We provide additional insights into the novel mechanisms possibly underlying the association between OH and molecular pathways related to endostatin. To date, drugs that increase vascular tone, such as midodrine and pyridostigmine, have been largely used to treat OH.^45,46^ The potential use of NO inhibition in the treatment of OH remains largely unexplored, although small clinical trials have shown promising results when administering intravenous infusions of NO synthase inhibitors in patients with OH^47^ and to treat orthostatic intolerance in patients with primary autonomic failure.^41^ Larger prospective studies are needed to investigate the role of NO synthase inhibition in OH, as well as examining alternative modes of administration that may facilitate the utility of this class of medication for the treatment of OH.

### Strengths and limitations

To our knowledge, this is the first observational study to investigate an association between OH and endostatin. It provides novel insights into the relationship between circulating endostatin and cardiovascular autonomic dysfunction. The robustness of the study is further enhanced by strict adherence to a standardized examination protocol for all participants, which included beat-to-beat haemodynamic monitoring. This rigorous approach significantly reduces the likelihood of inaccurate diagnoses or undetected cases of OH, thereby ensuring the reliability of our findings in establishing this association.

While our study provides relevant insights, its scope and impact are limited by its single-centre and observational design. Such a framework inherently introduces selection and referral biases, which significantly constrain the generalizability of our findings. Additionally, the cross-sectional design prevents conclusions about causality. The control group consisted of subjects referred to the syncope unit with prior history of orthostatic intolerance and/or syncope but negative HUT. Although we aimed for an equal number of plasma samples, i.e. 160 controls and 160 OH patients, unfortunately, 14 plasma samples for OH patients and 10 plasma samples for controls were excluded due to problems with the immunoassay and insufficient sample volumes. Moreover, the optimal predictive cut-off value for endostatin identified through our ROC analysis in *Figure 3* serves merely as an initial reference point for future prospective cohort studies, as our aim was not to use endostatin as a diagnostic marker for OH. The discriminative power for endostatin may vary depending on various clinical circumstances. Unfortunately, the manufacturer of the endostatin assay does not provide any reference ranges for healthy individuals. Thus, we have not provided any reference intervals for healthy vs. disease subjects. In our study, only two OH patients had a history of Parkinson’s disease. Unfortunately, we lacked information on other primary neurogenic causes of OH, such as primary autonomic failure and multiple system atrophy. Given these constraints, while plasma endostatin is a promising biomarker, the evidence is insufficient for clinical application. External validation through multicentre, longitudinal, cohort studies is essential to confirm our findings and establish the clinical utility of endostatin measurements.

## Conclusions

Patients with OH have increased circulating levels of endostatin, independent of age, sex, prevalent cancer, cardiovascular disease, and traditional cardiovascular risk factors. Our results highlight the relevance of investigating this molecular pathway in relation to cardiovascular risk mediated by the presence of OH. Further studies are warranted to assess the prognostic role of endostatin in individuals with OH and its possible relationship with other cardiovascular risk markers.

## Lead author biography


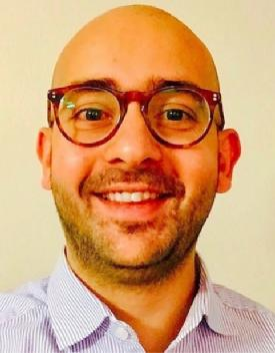


Fabrizio Ricci, MD, PhD, MSc, is a clinical cardiologist and assistant professor at the Department of Neuroscience, Imaging, and Clinical Sciences, ‘G.d'Annunzio’ University of Chieti-Pescara, Italy. He has focused his research on the field of cardiovascular imaging and on the assessment of cardiovascular autonomic dysfunction covering broad aspects of syncope and orthostatic intolerance with a focus on underlying mechanisms, biomarkers, treatment, and prognostic implications. This activity is testified by a valid scientific production and participation as a speaker at national and international conferences. Currently, he also collaborates with the Cardiovascular Research Unit of the Department of Clinical Sciences, Lund University, Malmö, Sweden.

## Supplementary Material

oeae030_Supplementary_Data

## Data Availability

Anonymized data that support the findings of this study are available from the corresponding author upon reasonable request.
