# Supplementary material for: Orthostatic hypotension is associated with higher levels of circulating endostatin
Source: Eur Heart J Open. 2024 Apr 10;4(3):oeae030. doi: 10.1093/ehjopen/oeae030 (PMC11068211; doi:10.1093/ehjopen/oeae030)
Supplement: oeae030_Supplementary_Data [file oeae030_supplementary_data.docx]

**Supplement**

**Supplementary Table S1.**

**Multiple linear and non-linear regression analysis of the individual effect of orthostatic systolic blood pressure (SBP) drop on circulating endostatin levels adjusted for baseline supine SBP, age, and antihypertensive treatment.**

| **Model** | **R2 value** | **Term** | **Coefficient** | **Standard Error** | **t-value** | **p-value** |
| --- | --- | --- | --- | --- | --- | --- |
| **Linear** | 0.24 | Intercept | -13.84 | 2.1 | -6.59 | <0.001 |
|  |  | Endostatin | -0.00023 | 0.00005 | -4.6 | 0.025 |
|  |  | Supine SBP | -0.358 | 0.047 | -7.62 | <0.001 |
|  |  | Age | -0.232 | 0.069 | -3.36 | 0.001 |
|  |  | Antihypertensive treament | -1.78 | 0.35 | -5.09 | <0.001 |
| **Non-Linear** | 0.27 | Intercept | 43.48 | 5.25 | 8.28 | <0.001 |
|  |  | Endostatin | -0.00014 | 0.00003 | -4.67 | 0.034 |
|  |  | Supine SBP | -0.381 | 0.058 | -6.57 | <0.001 |
|  |  | Age | -0.245 | 0.072 | -3.4 | 0.002 |
|  |  | Antihypertensive treament | -1.8 | 0.38 | -4.74 | <0.001 |

The coefficients in the linear model suggest significant effects of all predictors with a moderate model fit of the model R2=0.24, explaining 24% of the variance in orthostatic systolic blood pressure (SBP) drop. The non-linear model provides slightly improved model fit with an R2 of 0.27, explaining 27% of the variance in orthostatic SBP drop.

**Supplementary Figure S1.**

**Multiple linear regression analysis of the individual effect of orthostatic systolic blood pressure (SBP) drop on circulating endostatin levels adjusted for baseline supine SBP, age, and antihypertensive treatment.**

**
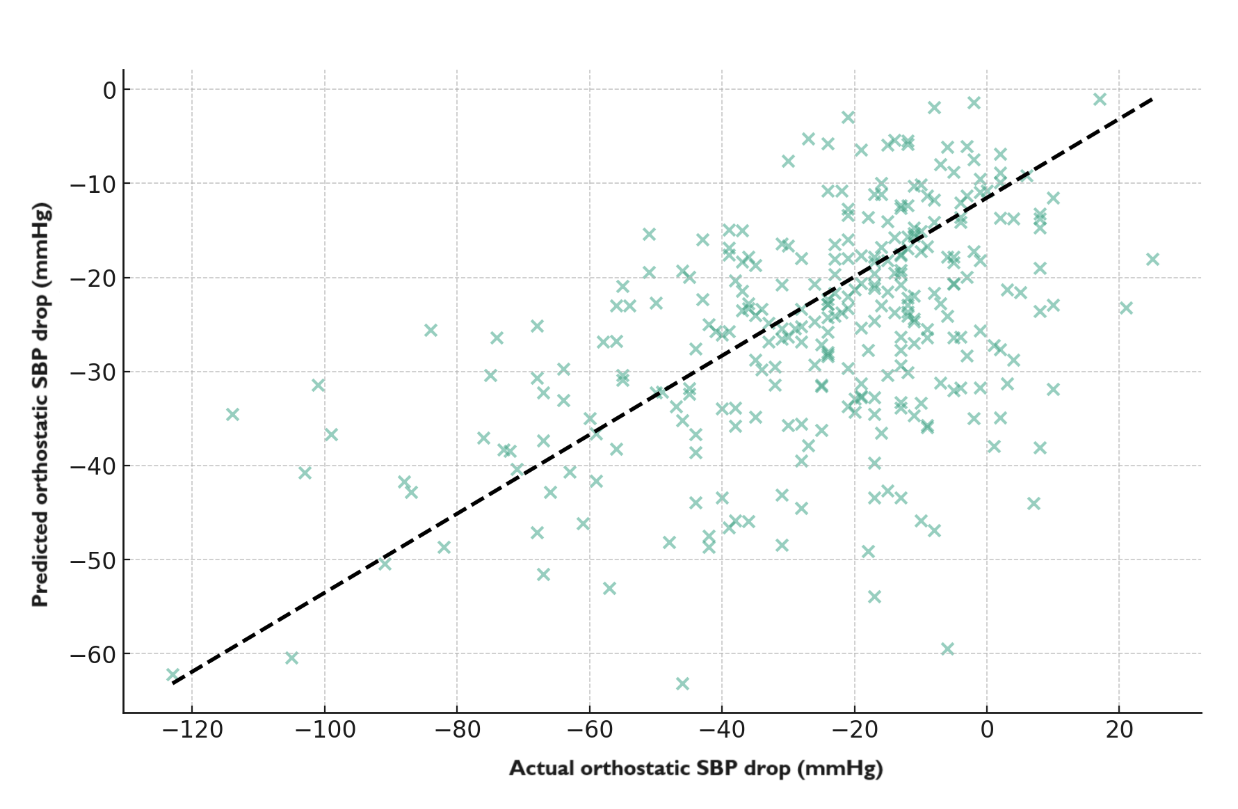
**

The plot illustrates the actual vs. predicted systolic blood pressure (SBP) drop using the extended linear regression model. The dashed line represents the perfect prediction, whereas the scatter of points around this line indicates the model's accuracy in predicting orthostatic SBP drop, with closer points representing a better fit.

**Supplementary Figure S2.**

**Multiple non-linear regression analysis of the individual effect of orthostatic systolic blood pressure (SBP) drop on circulating endostatin levels adjusted for baseline supine SBP, age, and antihypertensive treatment.**

**
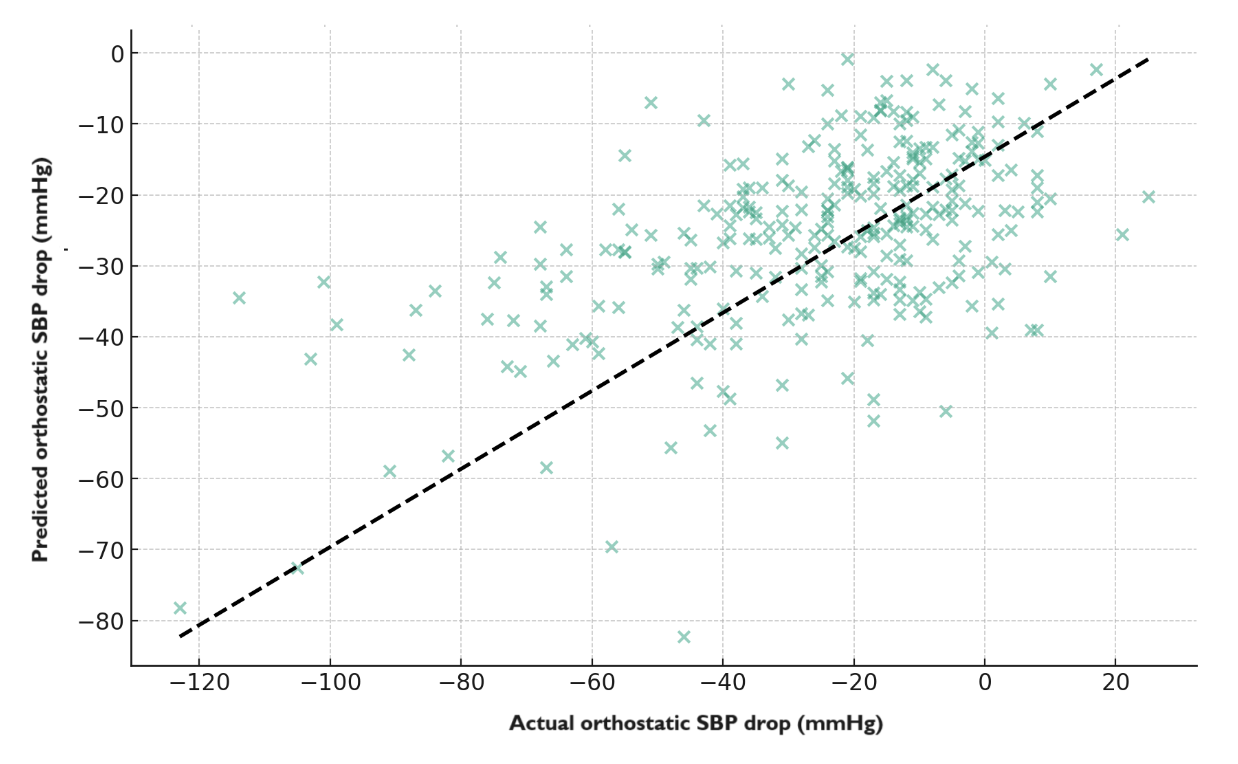
**

The plot illustrates the actual vs. predicted systolic blood pressure (SBP) drop using the extended non-linear regression model. The dashed line represents the perfect prediction, whereas the scatter of points around this line indicates the model's accuracy in predicting orthostatic SBP drop, with closer points representing a better fit.

**Supplementary Figure S3.**

**Interaction effect between baseline supine systolic blood pressure (SBP) and circulating endostatin levels on predicted orthostatic SBP drop.**


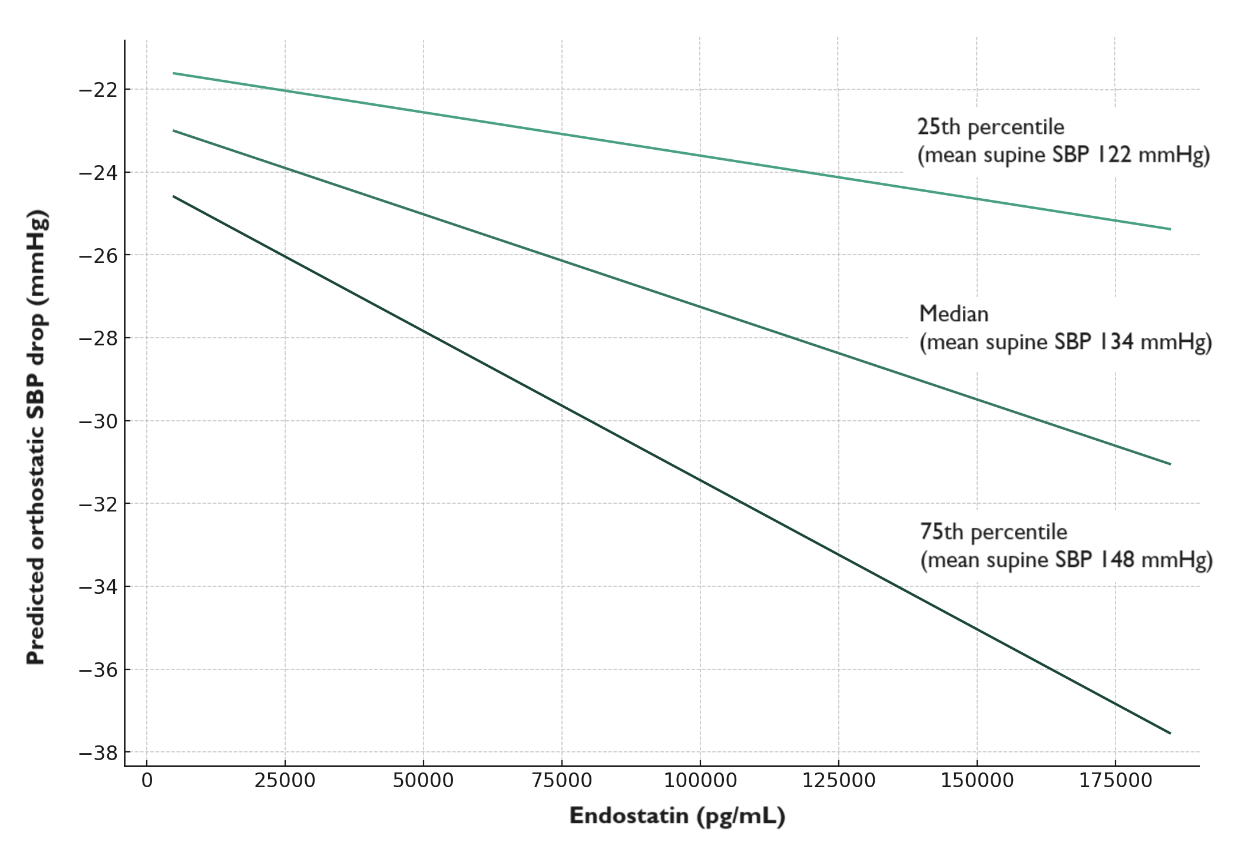


The plot illustrates the interaction effect between circulating endostatin levels and baseline supine systolic blood pressure (SBP) on predicted orthostatic SBP drop (P for interaction 0.046). The lines represent the model's predictions across different ranges of endostatin levels stratified according to the 25^th^, median, and 75^th^ percentiles of mean baseline supine SBP levels. The slope appears steeper for higher baseline supine SBP values, suggesting a stronger relationship between increasing endostatin levels and orthostatic SBP drop at higher baseline SBP levels.
